# The Neurodevelopmental Dynamics of Multilingual Experience During Childhood: A Longitudinal Behavioral, Structural, and Functional MRI Study

**DOI:** 10.3390/brainsci15010054

**Published:** 2025-01-09

**Authors:** Pasquale Anthony Della Rosa, Gerda Videsott, Virginia Maria Borsa, Eleonora Catricalà, Nicolò Pecco, Federica Alemanno, Matteo Canini, Andrea Falini, Rita Franceschini, Jubin Abutalebi

**Affiliations:** 1 Department of Neuroradiology, Istituto di Ricovero e Cura a Carattere Scientifico (IRCCS), Ospedale San Raffaele, 20132 Milan, Italy; 2 “br-ing” Primary and Lower Secondary Bilingual School, Via San Tommaso, snc, Castelvenere, 82037 Benevento, Italy; 3 Faculty of Design and Art, Free University of Bozen-Bolzano, 39100 Bolzano, Italy; 4 Department of Human and Social Sciences, University of Bergamo, Piazzale S. Agostino, 2, 24129 Bergamo, Italy; 5 Istituto di Ricovero e Cura a Carattere Scientifico (IRCCS) Mondino Foundation, 27100 Pavia, Italy; 6 ICoN Cognitive Neuroscience Center, Institute for Advanced Studies, Istituto Universitario di Studi Superiori (IUSS), 27100 Pavia, Italy; 7 Neuropsychology Service, Department of Rehabilitation and Functional Recovery, San Raffaele Scientific Institute, Vita-Salute San Raffaele University, 20132 Milan, Italy; 8 Faculty of Medicine and Surgery, Vita-Salute San Raffaele University, 20132 Milan, Italy; 9 Language Study Unit, Free University of Bozen-Bolzano, 39100 Bolzano, Italy; 10 Centre for Neurolinguistics and Psycholinguistics (CNPL), Faculty of Psychology, Vita-Salute San Raffaele University, 20132 Milan, Italy; 11 Department of Language and Culture, The Arctic University of Norway, 9019 Tromsø, Norway

**Keywords:** multilingualism, childhood, longitudinal, neuroimaging, cognition

## Abstract

Background/Objectives: A neurobiological framework of bi- or multilingual neurocognitive development must consider the following: (i) longitudinal behavioral and neural measures; (ii) brain developmental constraints across structure and function; and (iii) the development of global multilingual competence in a homogeneous social environment. In this study, we investigated whether multilingual competence yields early changes in executive attention control mechanisms and their underlying neural structures in the frontal–striatal system, such as the dorsal anterior cingulate cortex/pre-supplemental area and the left caudate. Methods: We employed longitudinal neuroimaging and functional connectivity methods in a small group of multilingual children over two years. Results: We found that the dACC/preSMA is functionally influenced by changes in multilingual competence but not yet structurally adapted, while the left caudate, in a developmental stage, is influenced, adapts, and specializes due to multilingual experience. Furthermore, increases in multilingual competence strengthen connections between the dACC/preSMA, left caudate, and other structures of the cognitive control network, such as the right inferior frontal gyrus and bilateral inferior parietal lobules. Conclusions: These findings suggest that multilingual competence impacts brain “adaptation” and “specialization” during childhood. The results may provide insights and guide future research on experience-expectant and experience-dependent brain plasticity to explain the “interaction” between multilingualism and neurodevelopment.

## 1. Introduction

Early childhood represents a prominent period of neurodevelopment, during which dynamic environmental factors can profoundly shape cognitive trajectories through adaptations in brain structure and function [1,2,3]. This stage is characterized by significant maturation in brain regions associated with executive functions, including the prefrontal cortex and its subcortical connections, leading to increased efficiency in cognitive control processes like attentional shifting, inhibitory control, and working memory [4]. Moreover, this developmental window may constitute a sensitive period for experience-dependent neuroplasticity, where environmental factors such as bilingual experience can exert a particularly strong influence on shaping neurocognitive development [5,6].

Indeed, the protracted trajectory of executive function development continues throughout childhood and adolescence [7]. Between the ages of 8 and 10, children demonstrate ongoing refinement of inhibitory control, working memory capacity, and cognitive flexibility [8]. While earlier research focused on individual executive components, more recent studies emphasize the importance of coordinating these processes for complex cognitive tasks [9].

Bilingual children provide a unique lens into this developmental process as they face the challenge of simultaneously acquiring two linguistic systems [10]. This experience of managing dual languages has been shown to influence the development of executive functions, with bilingual children demonstrating advantages in tasks involving inhibitory control, attentional switching, and working memory, particularly under conditions of heightened cognitive demand [11,12].

The ongoing usage of multiple languages triggers continuous adaptations in both brain structure and function, referred to as “constant restructuring” [13,14]. Importantly, these neural adaptations are not limited to language-specific regions but extend to domain-general executive control areas as well. Neuroimaging studies in multilingual children have provided empirical evidence for such continuous adaptations, highlighting changes in brain structure and function that align with increased demands on attentional control and language processing. For instance, the study by Thieba et al. [15] investigated young children aged 3–5 years raised in multilingual versus monolingual environments using resting-state functional MRI and structural MRI. They found that children in multilingual environments exhibited increased functional connectivity between the left inferior frontal gyrus (IFG) and language/attention areas, suggesting an enhanced integration within the language network. Conversely, they displayed decreased connectivity to areas involved in the default mode and ventral attention networks, implying reduced influence from extraneous networks. No significant structural differences in the IFG were observed between groups, suggesting that functional alterations may precede structural changes in response to early multilingual exposure. Such findings suggest that the cumulative experience of managing multiple languages exerts a significant influence on the neural architecture underlying cognitive control.

Furthermore, the frontal–striatal system represents a pivotal neurodevelopmental target for structural and functional adaptation to a bi- or multilingual condition in childhood [13]. This system plays a dynamic role in attentional control by routing signals across cortical regions and the striatum, facilitating efficient task performance under conditions of high cognitive demand. Within the fronto-striatal system, the dorsal portion of the Anterior Cingulate Cortex (ACC) bordering the pre-supplementary motor area (preSMA) is particularly important for monitoring information and detecting errors as part of target-language preparation [16]. In contrast, the left caudate nucleus is believed to manage target-language selection during different stages of language use, owing to its involvement in conflict monitoring and resolution [17]. These regions together contribute to an integrated system for language control, emphasizing the neurodevelopmental importance of adaptive responses to multilingual contexts.

The present study aims to elucidate the dynamic neurobiological mechanisms by which multilingual experience shapes neurodevelopmental adaptations in the frontal–striatal system. In particular, we explore the hypothesis that variations in multilingual competence, defined as a comprehensive measure that captures an individual’s competence across multiple languages not influenced by broader academic performance [18,19], over a short-term period within a prominent neurodevelopmental window, may drive functional and structural changes in the brain to support efficient and optimal behavioral performance. Pilatsikas [20] posits that these adaptations are contingent on the nature and extent of children’s multilingual experiences, reflecting a neural adaptation effect in both experience-dependent and experience-expectant forms. Recent evidence suggests that multilingual competence exerts a dual influence, modulating neural activity and plasticity based on the readiness of the underlying brain structures [21,22,23,24,25].

To investigate these hypotheses, we employed a longitudinal design using structural and functional neuroimaging, capturing two measurement timepoints across a two-year period. Specifically, (1) we sought to determine how changes in behavioral measures of executive attentional control relate to the degree of global multilingual competence over time. We hypothesized that increased multilingual competence would correlate with improvements in attentional control; (2) we investigated how differences in the children’s functional brain activation in response to attentional control demands are modulated by changes in multilingual competence. We focused on the functional activation patterns in the dACC/preSMA and left caudate to assess whether multilingual experience enhances neural efficiency during executive tasks; (3) we examined the influence of multilingual competence-related behavioral performance on structural and functional adaptations in brain regions engaged in cross-language activation and language control. By linking changes in multilingual competence to adaptations in the frontal–striatal circuitry, we aimed to capture how dynamic environmental inputs shape neurodevelopment at both structural and functional levels; (4) we explored how short-term changes in multilingual competence interact with the development of functional coupling between key regions involved in executive control, including the ACC/preSMA and left caudate, with broader brain networks involved in language processing and executive control. This allowed us to capture the extent to which increased multilingual competence enhances complex cognitive functions across the brain, supporting both language and attentional control.

## 2. Materials and Methods

### 2.1. Participants

Sixteen healthy multilingual children with no history of psychiatric or neurological disorders participated in this follow-up fMRI study. All children were raised in the small, linguistically homogeneous region of South Tyrol, Italy, where German, Italian, and Ladin are commonly used in daily life. Within the participant group, the primary language (L1) was either German or Italian, learned within the family and school contexts, while the use of English and Ladin was primarily passive. The multilingual experiences of all children were assessed using three sociolinguistic measures: (1) self-rated linguistic proficiency, (2) self-reported language exposure, and (3) the amount of time and activities conducted in each language, as determined through a custom questionnaire completed by the participants. The sociolinguistic data presented in Table 1, including daily language use, exposure, and competence, were employed to ensure sample homogeneity and provide context about the participants’ multilingual backgrounds, enabling a clear interpretation of the relationship between multilingual competence and attentional outcomes.

Informed consent was obtained from all participants’ parents, and all children assented to their participation in the study. The present study was approved by the Central Research Committee of the Free University of Bozen-Bolzano (Project Code: BW 5034; Date of Approval: 11 September 2008) in accordance with the Helsinki Declaration.

- The table presents the group’s average number of hours spent using each language during the week and weekend for Daily Activities (minimum value = 0 h, maximum value = 20 h);
- The table includes the group average self-rated measures of language exposure and competence, which were collected as percentages and transposed to a 4-point Likert scale (above 90% = 4; 50–90% = 3; 10–50% = 2; below 10% = 1);
- The “Production” and “Comprehension” columns represent the participants’ self-rated proficiency levels for the different languages they speak, which are distinct from the general “Language Production” and “Language Comprehension” columns that focus on language exposure in different contexts;
- The languages spoken by the participants were classified from L1 to L4 based on the percentages of linguistic competence, exposure, and daily use in order to define the multilingual interactional context for each subject. L1 (German = 70%, Italian = 30%); L2 (German = 30%, Italian = 70%); L3 (English = 90%; Ladin = 10%); L4 = (Ladin = 90%; English = 10%).

### 2.2. Multilingual Competence and Attention Measures

To generate a more objective and specific measure of multilingual proficiency, we calculated a global multilingual competence score for each participant at both time points (T1 and T2). This involved subtracting the mean value of all subjects’ grades from the mean value of grades related to all language subjects. This corrected scoring method aimed to isolate the linguistic abilities of each child from their overall academic performance, accounting for individual differences in general scholastic aptitude. The rationale was to focus on language-specific skills rather than general cognitive capacities, as suggested by Franceschini [26] and applied in prior studies of multilingual competence [18,19].

During the fMRI sessions, participants completed the Attention Network Test [27], responding via right or left button presses to indicate the direction of a centrally presented arrow. The target arrow was flanked by stimuli pointing in the same or opposite direction. Given the study’s objectives, the analysis concentrated solely on the conflict effect, with 64 incongruent and 64 congruent trials administered at each scan.

### 2.3. MRI Data Acquisition

We utilized a 3-Tesla Achieva Philips MRI scanner (Philips Medical Systems, Best, NL) equipped with an 8-channel sense head coil to acquire the MRI data. The functional data were obtained using an echo-planar imaging sequence sensitive to blood-oxygen-level-dependent contrast (BOLD) (TR = 2400 ms, TE = 30 ms, FOV = 24 cm^2^, matrix size 128 × 128). For each participant at each scan time (i.e., T1 and T2), the functional data were collected in two scanning sessions of the Attention Network Test task, each lasting 9.00 min. To account for gradient instabilities, the first 5 volumes of each session were discarded. A total of 222 volumes were acquired for each session, and each functional brain volume comprised 30 axial slices with a resolution of 2 × 2 × 4 mm, covering the entire brain. This was followed by the acquisition of an axial high-resolution structural MRI image (150 slice T1-weighted image, TR = 8.03 ms, TE = 4.1 ms, resolution = 1 × 1 × 1 mm) at both scan times.

### 2.4. MRI Data Preprocessing

The functional images from both time points were preprocessed and analyzed using the Statistical Parametric Mapping software (SPM8) (r6313) (Wellcome Department of Cognitive Neurology, London, UK) (https://www.fil.ion.ucl.ac.uk/spm/). A three-step combined functional–structural preprocessing approach was implemented to enable a more precise evaluation of longitudinal functional and structural changes between the two time periods. In the first step, it was necessary to accurately coregister the fMRI images for each time point with the respective T1-weighted images aligned to the AC-PC plane. The individual functional datasets were then checked for artifacts through the application of the TSDiffana routines (https://sourceforge.net/projects/spmtools/ (accessed on 4 August 2023)). ArtRepair 4 [28] was used to identify and repair slices with significant artifacts and to eliminate extracerebral noise. Additionally, a slice time correction was applied to compensate for varying slice acquisition times, and the time-series image volumes at each time period were spatially two-pass realigned and unwarped.

Prior to normalizing the images using SPM8, we employed the ArtRepair 4 software again to mitigate residual fluctuations caused by significant head motion. This allowed us to identify volumes with considerable rapid scan-to-scan motion (1 SD change in the head position) or outliers relative to the global mean signal (3 SD from the global mean). Volumes exhibiting motion artifacts and outliers in relation to the global mean signal were excluded and replaced through interpolation of the 2 nearest non-repaired volumes. The interpolated volumes were then partially deweighted when calculating the first-level models on the repaired images [29]. Additionally, the time series were despiked to remove extreme motion and global signal amplitude outliers, and a brain tissue mask was generated by thresholding the mean time-series image. This mask was used to eliminate any remaining extracranial voxels. By implementing this rational process to pinpoint actual movement-related and global signal issues, we had to exclude the data for one participant due to excessive movement associated with poor behavioral performance in terms of mean accuracy percentage (i.e., session 1 = 40.62%; session 2 = 33.33%) during both ANT fMRI sessions, resulting in a final sample size of 15 participants.

In the second step, a longitudinal preprocessing approach was applied to the structural scans from the two time points (T1 and T2) for each participant. This approach, integrated into the Voxel-Based Morphometry toolbox (VBM8) (r445) (https://neuro-jena.github.io/software.html#vbm) and implemented using SPM8 running on MATLAB R2008a (Mathworks), involved the following steps:

For each participant, the scans from the two time points were first aligned to the AC–PC plane and then realigned and averaged to create a mean image. Then, the original aligned scans were realigned to this mean image, bias-corrected, and segmented. The segmentation procedure used prior tissue probability maps generated from the Template-O-Matic toolbox (TOM8) (r12) (https://neuro-jena.github.io/software.html#tom), with the average ages between T1 and T2 (mean = 9.86, SD = 1.44) and sex as defining variables, as the investigated population consisted of children. An adaptive maximum a posteriori technique [30], extended by the addition of partial volume estimation [31], was used to segment the images into three classifications (gray matter, white matter, and cerebrospinal fluid). The data were subsequently de-noised using a hidden Markov Random Field approach [32]. Finally, deformation parameters were calculated by registering the mean image to the Montreal Neurological Institute (MNI) space using the default normalization procedure in SPM8.

In the third and final step, the deformation parameters determined during the normalization of the mean structural image in step 2 were applied to the individual preprocessed EPI volumes from both time points (T1 and T2) for each participant. This step was necessary to transform the functional data into a common MNI space, as defined by the SPM8 software. Following this spatial normalization, the longitudinally “structural-match-warped” EPI images were smoothed using a 6.0 mm full-width at half-maximum Gaussian filter.

### 2.5. Effects of Global Multilingual Competence on Behavioral Conflict Effect Measures

To evaluate changes in multilingual competence and attentional performance over time, we normalized the gMC corrected scores and the conflict effect measures using sign-rank methods. This normalization approach accounted for non-normal distributions and individual baseline differences in the small sample by focusing on relative rank changes over time. This allowed for meaningful comparisons of changes between the two time points, T1 and T2. We then investigated the Pearson correlational relationship between sign-ranked normalized differences in the behavioral conflict effect measure (RT difference between incongruent and congruent trials) from T2 to T1 and the sign-ranked normalized changes in gMC corrected scores between T2 and T1.

### 2.6. Effects of Global Multilingual Competence on Brain Function

Voxel-wise general linear models were constructed for each participant to independently generate conflict effect contrast images at the two time points, T1 and T2. Event-related regressors representing incongruent and congruent flanker trials were modeled by convolving a delta function of each event type with the hemodynamic response function. Only trials with correct responses were included in the analysis. The data and model were high-pass filtered to a cutoff of 1/128 Hz. To assess the relationship between longitudinal change in multilingual competence and intraindividual conflict effect BOLD signal change, we correlated the gMC score change and BOLD conflict effect signal change between T2 and T1. For each participant, T2 minus T1 functional conflict contrast difference images were used in a one-sample t-test model with one covariate to identify regions with a positive and negative correlation between the BOLD conflict effect signal change and gMC change. The statistical analysis was restricted to voxels within a gray matter mask created from the mean of gray matter segmentations at both T1 and T2 using the SPM Masking toolbox [33]. The effects of the gMC score change covariate are reported. Correlations were considered significant if they reached a statistical threshold of *p* < 0.001 at the voxel level and *p* < 0.05 after correction for multiple comparisons (FWE) at the cluster level (k = 48 voxels).

### 2.7. Effects of Global Multilingual Competence on Brain Function and Structure

A multiple-level region of interest analysis was performed to determine the degree to which multilingual competence influenced the neural specialization of two regions involved in both executive attention and language control: the dorsal anterior cingulate cortex/pre-supplementary motor area (dACC/preSMA) complex and the left caudate (LC). This analysis aimed to trace the developmental trajectory of the effect of multilingual competence on attentional control in terms of structure, function, and the interplay between these two dominant regions of the frontal–striatal system. Notably, these two regions were selected a priori based on previous evidence [34,35,36,37,38,39,40] highlighting their roles in language and executive attention control. Spherical regions of interest were created, with a radius of 10 mm for the dACC/preSMA and 8 mm for the left caudate, using the Marsbar tool [41]. The center coordinates for the dACC/preSMA were x = 5, y = 15, z = 40. Marsbar was then employed to extract the mean blood-oxygen-level-dependent signal time series in the dACC/preSMA for the functional conflict effect contrast at T1 and T2. Additionally, non-smoothed and modulated dACC/preSMA gray matter (GM) volumes in liters were extracted at both time points using the Easy_volume Utility (https://cpernet.github.io/index.html (accessed on 4 August 2023)) [42].

To obtain an unbiased estimate of BOLD activation and gray matter from the left caudate, we created an 8 mm radius spherical region of interest centered on the median of the x, y, and z coordinates (x = −8; y = 8; z= 8) reported for the LC in previous studies on bilingual language control [36,39,40,43]. We then extracted the mean BOLD signal of all voxels and GM volumes in liters within this LC-defining sphere at both T1 and T2, using the same procedure described for the dorsal anterior cingulate cortex/pre-supplementary motor area (dACC/preSMA) region.

Additionally, total GM volumes were calculated at both T1 and T2 to compute a total GM ratio difference score at T2. This score was obtained by subtracting the ratio between the individual subject’s GM total value at T1 and the median value of GM total values for the whole group (n = 15) at T1 from the same ratio measure at T2. This calculation provided an estimate of the total gray matter volume change over the two measurement points.

1. First, we assessed the relationship between dACC\preSMA and LC fCE estimates at T1, T2 and between T2 minus T1 fCE differential values by means of a Pearson Correlation analysis;
2. Second, we built a multiple regression model in which the behavioral CE difference between T1 and T2 was the dependent variable while fCE differential values between T1 and T2 for dACC\preSMA, for LC and gMC differential values between T1 and T2 were independent variables to test if this model predicted RT differences between T1 and T2 for the behavioral CE to a statistically significant degree;
3. Third, we employed a hierarchical multiple regression approach to assess the unique relationship between changes in global multilingual competence (gMC) and neural structural and functional “adaptation” in the dorsal anterior cingulate cortex/pre-supplementary motor area (dACC/preSMA) or left caudate between the two time points, T1 and T2. Specifically, we constructed two separate hierarchical regression models to investigate any specific effects of multilingual competence on the structural development of each region that were associated with the observed differences in BOLD signal functional conflict effect (fCE) estimates between T1 and T2. In each model, the fCE differential values between T1 and T2 served as the dependent variable. In the first step, we included the total gray matter increase between T1 and T2 as an independent variable. We then added the area-specific GM difference between T1 and T2 and the gMC differential values between T1 and T2 in the second step. Finally, we introduced the interaction term, calculated as the cross-product between the area-specific GM differences and gMC differences, in the third step. For each step of the regression, we assessed the significance of the changes in R-squared, and if a block produced a significant change, we examined the beta weights to determine the individual contributions of the variables.

### 2.8. Effects of Global Multilingual Competence on Brain Functional Connectivity

To evaluate the impact of multilingual competence on functional brain connectivity underlying complex attentional and language control networks at a whole-brain level over time, we examined the specific effect of changes in global multilingual competence between the two time points (T1 and T2) on the functional connectivity patterns of the dorsal anterior cingulate cortex/pre-supplementary motor area (dACC/preSMA) and left caudate in terms of functional conflict effect (fCE) BOLD activity measured at T2. Specifically, we mapped the differential functional interplay between the dACC/preSMA and LC with other brain regions as modulated by the difference in global multilingual competence between T1 and T2. A task-based functional connectivity analysis was conducted in MATLAB using the CONN toolbox (version 14.n) ((http://www.nitrc.org/projects/conn) [44] to explore how variations in multilingual competence over time can shape brain functional connectivity patterns of language and executive control regions in response to conflict.

At the second time point (T2) for each participant, the CompCor method [45] was employed within the CONN software to identify principal components associated with segmented white matter and cerebrospinal fluid. Additionally, individual participants’ motion parameters and the main effects of task conditions were included as confounds in a first-level analysis to mitigate the impact on intrinsic functional connectivity or the measurement of simple task-related co-activation. The event-related fMRI data, which had been normalized and smoothed, were not subjected to band-pass filtering, and the default “hrf” temporal weights were maintained. Subsequently, a seed-to-voxel analysis was conducted to explore the modulation of global multilingual competence (gMC) difference measures on functional conflict effect (fCE) connectivity patterns at T2 for (1) the dorsal anterior cingulate cortex/pre-supplementary motor area (dACC/preSMA) and (2) the left caudate with other brain regions.

The a priori selected seed regions were as follows: (i) the bilateral dorsal anterior cingulate cortex (i.e., BA 32 left and right), derived from the Tzourio-Mazoyer template [46], to represent the dACC/preSMA; and (ii) a normalized and resliced mask for the left caudate, computed using the WFU PickAtlas (r3.0.5) toolbox (https://www.nitrc.org/projects/wfu_pickatlas) [47], to represent the LC seed. Temporal correlations were computed between the mean time course across voxels within each seed and all other voxels in the brain. Seed-to-voxel connectivity estimations were obtained by calculating the correlation between the signal from each seed and the signal at every brain voxel, using the incongruent and congruent conditions at T2 as the temporal variable.

Whole-brain functional connectivity analyses were conducted to investigate the impact of changes in global multilingual competence on the functional interplay of key language and executive control regions. Specifically, a seed-to-voxel approach was used to map modulations in functional connectivity patterns associated with the dorsal anterior cingulate cortex/pre-supplementary motor area and left caudate as a function of the difference in global multilingual competence between the two time points. The statistical analyses involved a second-level group analysis that included the change in multilingual competence as a between-subjects covariate. This allowed for the identification of brain areas exhibiting significant connectivity changes with the a priori seed regions, as driven by the multilingual competence differences. The reported results met a statistical threshold of *p* < 0.001 at the voxel level and *p* < 0.05 FDR-corrected at the cluster level, with all coordinates referring to peak activations in standard MNI space.

## 3. Results

### 3.1. Effects of Global Multilingual Competence on Behavioral Conflict Effect Measures

The data showed high accuracy rates, exceeding 95%, in both the incongruent and congruent conditions. At the first time point (T1), the mean percentage accuracy was 96.39%, which increased to 98.51% at the second time point (T2). Reaction times for incongruent and congruent flanker trials were screened, and those falling outside two standard deviations of the mean were excluded. The mean behavioral conflict effect decreased from 147 ms at T1 to 124 ms at T2.

Sign-ranked normalized differences in the behavioral conflict effect measure and the global multilingual competence (gMC) scores between T2 and T1 were calculated and rescaled to a common range of −1 to +1. Positive difference values in multilingual competence indicated increased proficiency, while positive difference values in conflict effect measures reflected improved attentional control.

**Figure 1:**
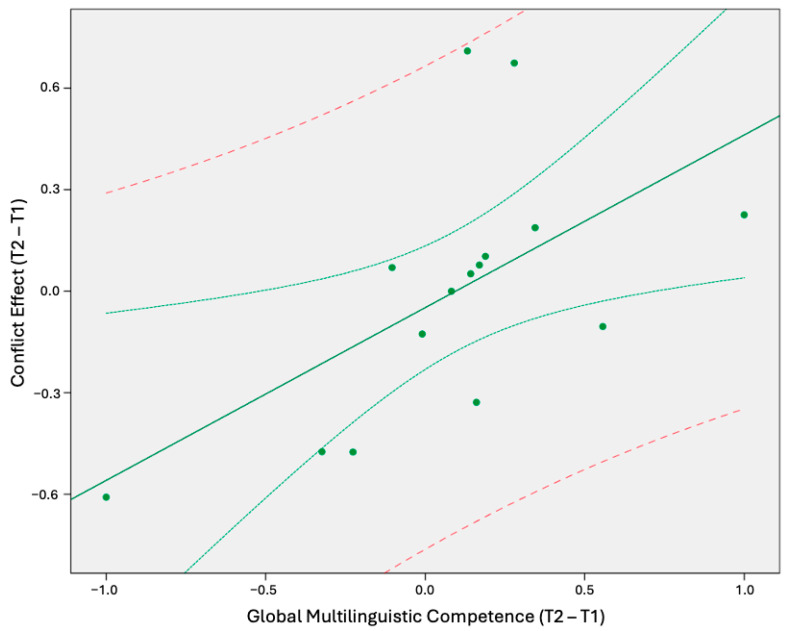
Figure 1 illustrates the relationship between the sign-rank normalized differences in global multilingual competence (gMC) and the conflict effect measures from the first time point (T1) to the second time point (T2). The *x*-axis represents the difference in gMC scores between T1 and T2, which were rescaled and transposed on a range from −1 to +1. The *y*-axis depicts the corresponding difference in CE values between T1 and T2, similarly rescaled. The final CE difference value quantifies the change in the conflict effect measure between the two time points, accounting for the rank of the CE at T1 and T2. Notably, a positive difference value in multilingual competence corresponds to an overall positive change in the CE from T1 to T2. The green line represents the regression line fitted to the data with mean (green dotted line) and 95% (red dashed line) confidence intervals. The green dots correspond to the position of subject data points (n = 15) and their corresponding values for the variables represented on the x-axis and y-axis.

Pearson Correlation analyses revealed a significant positive linear relationship (r = 0.587, *p* = 0.021) between changes in gMC and the differential values of the conflict effect between the two time points (see Figure 1).

### 3.2. Effects of Global Multilingual Competence on Brain Function

**Figure 2:**
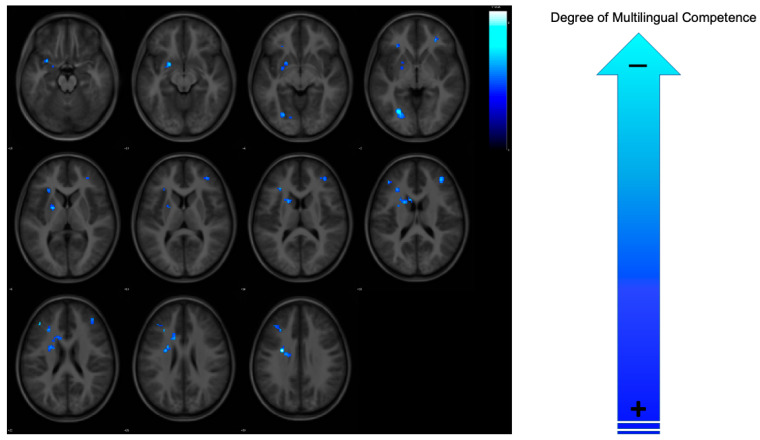
A group-level statistical analysis revealed brain regions where a significant negative correlation occurred between conflict effect-related BOLD activity and changes in global multilingual competence between the two time points (T1 and T2). These findings were thresholded at a cluster-level significance of *p* < 0.05, family-wise error corrected.

The fMRI correlation analyses revealed brain regions exhibiting a negative association, where increased conflict effect-related neural activity was coupled with a decline in global multilingual competence between the two time points (see Figure 2 and Table 2). Specifically, a negative correlation was found between change in global multilingual competence and brain activation in the left caudate, dorsolateral prefrontal cortex (extending to the pars triangularis of the inferior frontal gyrus), left putamen, left occipital fusiform gyrus, and right dorsolateral prefrontal cortex. This suggests that participants demonstrating negative changes in global multilingual competence over time displayed heightened neural responses in both language control and general executive control regions. Conversely, no significant positive correlations were observed, where decreased conflict effect-related neural activity between the two time points was linked to positive changes in global multilingual competence.

### 3.3. Effects of Global Multilingual Competence on Brain Function and Structure

First, we examined the relationship between the functional conflict effect estimates in the dorsal anterior cingulate cortex/pre-supplementary motor area and the left caudate at both the first and second time points. Our analysis revealed no significant correlation between these regions at the initial time point (r = 0.364, *p* = 0.182). However, a significant correlation was observed at the second time point (r = 0.724, *p* = 0.002). Furthermore, we found a significant correlation between the differential changes in functional conflict effect estimates from the second time point to the first for the dorsal anterior cingulate cortex/pre-supplementary motor area and left caudate (r = 0.760, *p* = 0.001).

Second, the overall multiple regression model examining the differential values of functional conflict effect (fCE) between the two time points (T1 and T2) for the dorsal anterior cingulate cortex/pre-supplementary motor area (dACCpreSMA), the left caudate, and the global multilingual competence (gMC) differential values, in predicting the reaction time differences of the behavioral conflict effect between T1 and T2, was statistically significant (F(3,11) = 4,257, *p* = 0.032 (R2 = 0.53)).

**Figure 3:**
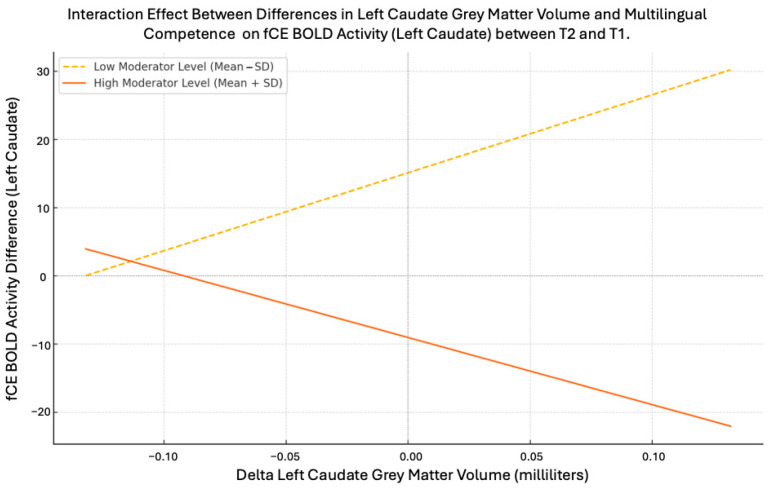
Positive values for fCE (functional conflict effect) correspond to a significant increase in BOLD activity in the left caudate between T1 and T2 in response to the conflict effect, while negative values correspond to a significant decrease in activity (*y*-axis) for varying levels of structural changes in the left caudate between T1 and T2 (i.e., delta gray matter) (*x*-axis) based on low or high differences in global multilingual competence between T1 and T2 (i.e., delta multilingual competence). The dashed line represents participants with low multilingual competence changes (mean − SD) (i.e., participants with multilingual competence changes that are one standard deviation below the average change, indicating below-average improvement), whereas the solid line represents participants with high multilingual competence changes (mean + SD) (i.e., participants with multilingual competence changes that are one standard deviation above the average change, indicating above-average improvement).

Third, for the dACCpreSMA, the first step of the hierarchical regression was not significant (R2 = 0.011; F(1,13) = 0.141, *p* = 0.713), while the second step was significant (R2 change = 0.430, F change (2,11) = 4.225, *p* = 0.043), and the third step was not significant (R2 change = 0.005, F change (1,10) = 0.081, *p* = 0.781). This indicates that the difference in global multilingual competence at the second step contributed significantly to the prediction of fCE differential values ((β) = −0.499, t-value = −2.207, *p* = 0.049). This suggests that multilingual competence significantly modulates the dACCpreSMA fCE activity between the two time points. For the left caudate, neither step 1 nor step 2 of the regression analysis yielded a significant result (step 1: R2 = 0.045; F(1,13) = 0.615, *p* = 0.447; step 2: R2 change = 0.219, F change (2,11) = 1.637, *p* = 0.239), whereas step 3 significantly contributed to the prediction (R2 change = 0.257, F change (1,10) = 5.372, *p* = 0.043, β = −1.747). The interaction analysis revealed that the relationship between structural changes in gray matter and functional BOLD activity in the left caudate was moderated by changes in multilingual competence (see Figure 3). Specifically, the effect of differences in global multilingual competence (either lower or higher than one standard deviation above the average change) on the conflict effect-related BOLD activity difference in the left caudate between the two time points significantly varied as a function of the magnitude of gray matter changes observed in this region. The plotted lines in Figure 3 represent the relationships for participants with lower versus higher multilingual competence changes. Positive values indicate an increase in BOLD activity related to the conflict effect, while negative values indicate a decrease, depending on the level of structural changes observed.

Table 3 includes the mean, standard deviation, and range for the variables analyzed in the regression model for the Left Caudate. The interaction effect shows that the relationship between changes in Left Caudate Gray Matter Volume (Delta Gray Matter Left Caudate (milliliters) (T2 − T1)) and Delta BOLD Activity for the conflict effect (Delta fCE BOLD Activity (T2 − T1)) is significantly moderated by changes in multilingual competence (i.e., Delta Multilingual Competence (T2 − T1)). The slopes for “Low Moderator Level” (Mean − SD) and “High Moderator Level” (Mean + SD) depict significant divergence in the relationship and are shown in Figure 3.

### 3.4. Effects of Global Multilingual Competence on Brain Functional Connectivity

**Figure 4:**
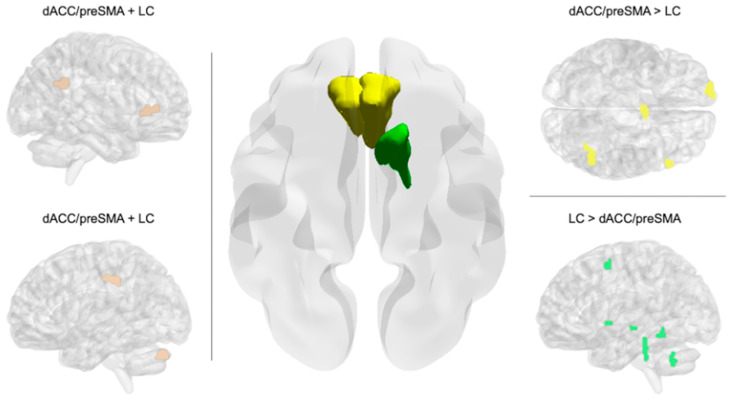
Center: The seed volumes for the dorsal anterior cingulate cortex/pre-supplementary motor area (dACC/preSMA) (yellow) and left caudate (green) have been smoothed and mapped to a standard template brain surface using BrainNet Viewer, shown in an elevated axial view from bottom to top. Left: The connectivity pattern (light brown) of the dACC/preSMA-LC complexhas been rendered on sagittal anatomical standard brain views using BrainNet Viewer [48] with partial transparency to convey depth information. This connectivity pattern is modulated by differences in multilingual competence between the two time points (T1 and T2). Right: Areas exhibiting stronger connectivity with the dACC/preSMA seedcompared to the LC seed (upper part) (yellow), as well as vice versa (lower part) (green), have been rendered on axial and sagittal views. These connectivity differences are induced by changes in global multilingual competence.

Increases in multilingual proficiency between the two time points significantly modulated the interplay between dorsal anterior cingulate cortex/pre-supplementary and left caudate combined seed-activity, with the left supramarginal gyrus, left cerebellum, and right inferior frontal gyrus extending to the insular cortex and angular gyrus in the right hemisphere, resulting in stronger functional connectivity (see Figure 4, left panel, and Table 3). Positive changes in multilingual competence between the two time points induced significantly stronger connectivity between the dorsal anterior cingulate cortex/pre-supplementary motor area seed and the left dorsolateral prefrontal cortex extending to the medial portion of the superior frontal gyrus, the right dorsolateral prefrontal cortex peaking in the pars triangularis of the inferior frontal gyrus, the right angular gyrus, and the right pre-supplementary motor area/supplementary motor area border, compared to the left caudate seed (see Figure 4, right panel, upper part, and Table 4). At a stringent FDR-clusterwise corrected threshold, the left caudate seed showed no significantly stronger connections with other brain areas modulated by increases in multilingual competence compared to the dorsal anterior cingulate cortex/pre-supplementary motor area seed. However, at a more lenient threshold (i.e., *p* < 0.001 uncorrected at both voxel and cluster levels) and for exploratory purposes, significantly stronger connections were observed for the left caudate seed with respect to the dorsal anterior cingulate cortex/pre-supplementary motor area seed, including the left fusiform gyrus extending to the posterior division of the inferior temporal cortex, the left hippocampus, the posterior division of the left parahippocampal gyrus, the left putamen, the left middle/superior frontal gyrus, and the right cerebellum (see Figure 4, right panel, lower part, and Table 4).

## 4. Discussion

In this study, multilingual children underwent two functional and structural neuroimaging sessions over a two-year interval, during which they completed the Attentional Network Task. The findings reveal a significant positive relationship between changes in multilingual competence and changes in executive attentional control over the two-year period. Specifically, children who demonstrated substantial gains in global multilingual proficiency also exhibited corresponding improvements in executive attentional performance, as reflected by a positive change in the conflict effect from the first to the second time point.

Additionally, we observed a negative relationship, where a decrease in global multilingual competence between the two time points was associated with an increased BOLD signal change related to the conflict effect. Specifically, smaller changes in multilingual proficiency were significantly coupled with greater functional engagement in a network of brain regions, primarily encompassing left frontal and subcortical areas as well as the right dorsolateral prefrontal cortex, which are known to support interference control and conflict resolution processes [16,49,50,51].

A decline in multilingual competence over time may stem from a shift in the balance between two or more languages, leading to a decreased ability to manage cross-linguistic activity and language control [52]. Existing studies on bilingualism have shown that increased brain activation during incongruent nonverbal stimuli or language-switching tasks [39,53,54,55] indicates the recruitment of specific brain regions to support the cognitive processing of interest [56], while decreased brain activity may reflect more efficient recruitment of brain functions [39,56]. Thus, it is crucial to investigate the brain–behavior relationship by integrating behavioral performance, brain functional activation, and brain structure plasticity [57,58].

Finally, we employed a three-step analysis procedure to uncover the association among these factors.

In the first step, we investigated the functional relationship between brain activity in the dorsal anterior cingulate cortex/pre-supplementary motor area (dACC/preSMA) and the left caudate during the executive attention control condition of the Attentional Network Task at each time point. We also examined the correlation between the difference in functional connectivity estimates (fCE) for these two regions across the two time points. This was performed to determine whether executive attention control mechanisms evoked by nonverbal stimuli are grounded in the cross-talk between activity in these frontal–striatal regions at any given time point and whether changes in processing interference between the two time points are captured by the functional relationship between dACC/preSMA and LC.

The findings indicate that there was no significant correlation between functional connectivity estimates (fCE) for the left caudate and dorsal anterior cingulate cortex (dACC) at the first time point (T1). However, a significant positive correlation was observed between the activity in these two regions at the second time point (T2), as well as in the difference in fCE estimates between the two time points. This suggests that a functional relationship between these two structures emerged only at T2 and appears to be sensitive to the functional adaptation of executive attention control mechanisms occurring between T1 and T2. In other words, these results imply that these two regions may begin to form part of a network responsible for managing cognitive control, and the efficiency of this network may be influenced by changes in multilingual competence over a two-year period [59,60,61].

Therefore, in the second step, we aimed to establish a “functional” association between brain activity and behavioral performance. We examined the differences observed between the two time points (T1 and T2) in measures of the behavioral conflict effect and three factors: (1) T2-minus-T1 differences in functional connectivity estimates (fCE) for the dorsal anterior cingulate cortex/pre-supplementary motor area (dACC/preSMA), (2) T2-minus-T1 differences in fCE for the left caudate, and (3) T2-minus-T1 differences in global multilingual competence measures. We verified that these predictors, when incorporated together in a regression model, were able to significantly predict the differences in conflict effect reaction times between T2 and T1. This model, which includes the differences in ACC and LC functional connectivity as well as the differences in multilingual competence between time points, explains the variation observed in the behavioral conflict effect between T2 and T1. In other words, the differences in activity in these two brain regions, combined with the differences in multilingual proficiency, can significantly inform our understanding of behavioral attentional control performance. This establishes a developmental link between executive attention control, the functional configuration of structures involved in both domain-general and language control, and a measure of multilingual experience [62].

In the third and final step, we examined the neural “specialization” of the dorsal anterior cingulate cortex/pre-supplementary motor area (dACC/preSMA) and left caudate to determine if both the functional and structural properties of these regions exhibit neural adaptation effects in response to differences in multilingual experience [24,63,64].

For the dACC/preSMA, we found that global multilingual competence modulates the functional activity differences between the two time points in a manner consistent with patterns observed in adults [65,66]. Specifically, a smaller difference in functional activity between the two time points was paralleled by greater positive differences in multilingual competence scores, independent of any specific structural changes occurring in this region during the same period. In other words, the functional activation differences associated with varying levels of multilingual competence do not change across differences in structural brain plasticity observed in the dACC/preSMA. This suggests that the dACC/preSMA may initially be involved in a range of functions, and the influence of multilingual competence on structural adaptation in this region may emerge later in development, following more prolonged experiences of bi-/multilingualism [37,67,68].

On the other hand, for the left caudate region, it appears that differences in multilingual competence between the two time points interact with its functional resources, modulating responsiveness to the cognitive processes underlying nonverbal executive attention control between the two time points. This relationship, however, varies depending on the degree of structural adaptation occurring within the LC. Specifically, for individuals displaying low LC structural plasticity, there was no significant increase or decrease in activity levels between the two time points, and the changes in functional connectivity estimates (T2-minus-T1) overlapped substantially between children with low and high differences in global multilingual competence (please see Figure 3). In contrast, for individuals exhibiting high LC structural plasticity, there was a significant difference in the change in functional connectivity estimates (T2-minus-T1) between children showing small versus large differences in global multilingual competence. This finding indicates a substantial increase in activity differences for children with minimal changes in multilingual competence, contrasted with a significant decrease in activity for those with large increases in multilingual competence (please see Figure 3). In summary, multilingual competence appears to be responsible for adapting the functional activity of the left caudate to meet the demands of executive attention control processing, particularly when structural changes within this region have already occurred [69,70].

These findings suggest that multilingual competence may drive a “dual” neural adaptation effect, where it shapes brain function and structure in both experience-dependent (i.e., low structural plasticity) and experience-expectant (i.e., high structural plasticity) ways [71]. This idea aligns with the Dynamic Restructuring Model proposed by Pliatsikas [13,19], indicating that multilingual competence “interacts” with functional activity and structural plasticity in a manner that varies depending on the developmental maturity of the underlying neural architecture [19,23].

In contexts where the brain’s structural components are not yet fully developed, multilingual proficiency appears to drive functional adaptations that optimize cognitive performance (i.e., experience-dependent plasticity). These functional adjustments are characterized by modulating activity in brain regions responsible for executive attentional control, such as the dorsal anterior cingulate cortex and pre-supplementary motor area. This suggests that early exposure to multiple languages can promote the development of cognitive flexibility through heightened functional plasticity. Conversely, when the underlying brain structures have already matured functionally, multilingual competence modulates functional activity in an experience-expectant manner, balancing functional demands more efficiently across varying levels of multilingual proficiency. This efficient utilization of functional resources is indicative of a more stable, mature neural system that has adapted to the linguistic and attentional control requirements of multilingualism, resulting in reduced overall energy expenditure [35–72].

In this framework, we infer that when executive attentional processes require specific functional resources and multilingual proficiency is relatively limited, the brain’s structural systems adapt by increasing functional responsiveness to meet cognitive demands. This adaptive mechanism enables functional activity to rise to the occasion, optimizing performance under conditions of emerging multilingual competence. Such adaptations involve the recruitment of additional neural circuits, particularly within the prefrontal cortex and subcortical regions, to support attentional control and language processing [73]. This recruitment process reflects a compensatory response, wherein the brain dynamically engages available resources to manage the increased cognitive load, thereby enhancing overall cognitive performance in multilingual contexts [74].

Conversely, if the neural structures are already functionally equipped, multilingual competence enables a more efficient distribution of functional resources, reflective of a more stable, experience-expectant response. This differentiation between adaptive functional responsiveness and experience-expectant modulation underscores the dynamic interplay between multilingualism and neurodevelopment. It suggests that the brain’s response to multilingualism is contingent on the developmental stage and existing structural characteristics of the individual [21,75]. As multilingual experience accumulates, the brain transitions from heightened functional adaptation to greater efficiency and specialization, indicative of the consolidation phase of neurodevelopment [76,77,78,79].

Our findings regarding the dorsal anterior cingulate cortex/pre-supplementary motor area (dACC/preSMA) and left caudate align with this hypothesis. On the one hand, the dACC/preSMA appears to be influenced by changes in multilingual proficiency but has not yet structurally adapted to the same extent as other regions involved in language control. This ongoing adaptation in the dACC/preSMA suggests that these areas are still undergoing functional reorganization, attempting to meet the demands of increased multilingual usage. On the other hand, the left caudate exhibits signs of structural adaptation and specialization, consistent with the consolidation phase of the Dynamic Restructuring Model, where subcortical regions increasingly support the efficiency of executive functions in multilingual individuals. The structural changes observed in the LC align with the DRM’s characterization of subcortical involvement during the consolidation stage, indicating that while the dACC/preSMA may still be undergoing functional adaptations, the LC is progressing toward structural specialization. This structural adaptation in the LC likely contributes to more automatic and efficient language control, reflecting the brain’s ability to fine-tune subcortical circuits in response to the demands of multilingualism. Such specialization facilitates a more seamless integration of language control processes, reducing cognitive load during multilingual tasks and thereby enhancing overall linguistic proficiency.

The observed differentiation between the dorsal anterior cingulate cortex/pre-supplementary motor area and the left caudate underscores the necessity of considering both functional and structural aspects of neuroplasticity when examining the effects of multilingual proficiency. While functional adaptations may confer immediate benefits in terms of enhanced cognitive flexibility and attentional control, structural adaptations contribute to the long-term efficiency and stability of these processes. This dual adaptation mechanism highlights the complexity of multilingual-induced neuroplasticity, wherein brain regions can be at varying stages of adaptation depending on the nature and extent of multilingual experience. By examining both functional and structural changes, this study offers a more comprehensive understanding of how multilingualism shapes the developing brain, supporting the idea that neuroplasticity is a multifaceted and dynamic process shaped by ongoing language experience [20].

The functional connectivity analysis further supports our hypothesis by demonstrating that increases in multilingual competence strengthen the connections between the dACC/preSMA-LC complex and frontal regions involved in conflict resolution and target selection [13,78], as well as parietal regions associated with attention shifting and monitoring of conflicting information [8,79]. Our data further indicate that multilingual competence modulates structure-specific conflict effect (CE) functional connectivity between distinct components of the executive control network, namely between the dACC/preSMA and the frontal subcomponents of the executive control system and between the LC and the temporal and basal–temporal language areas. Specifically, the dACC/preSMA appears to be primarily involved in top-down monitoring geared toward target-language preparation, thereby supporting the prefrontal regions in the response selection process [78]. The left caudate (LC), as part of a network of areas responsive to linguistic stimuli, is influenced by multilingual experience in determining language membership of word forms. This bottom-up influence highlights the LC’s role in language selection and monitoring, aligning with previous findings that emphasize its role in subcortical language control in bilinguals [23,69].

The functional connectivity analysis further corroborates our hypothesis by demonstrating that increases in multilingual proficiency strengthen the connections between the dorsal anterior cingulate cortex (dACC)/pre-supplementary motor area (preSMA)–left caudate complex and frontal regions involved in conflict resolution and target selection [13,78], as well as parietal regions associated with attention shifting and monitoring of conflicting information [8,79]. Our data additionally indicate that multilingual competence modulates structure-specific conflict effect functional connectivity between distinct components of the executive control network: specifically, between the dACC/preSMA and the frontal subcomponents of the executive control system, and between the LC and the temporal and basal–temporal language areas. Notably, the dACC/preSMA appears to be primarily engaged in top-down monitoring aimed at target-language preparation, thereby supporting the prefrontal regions in the response selection process [80]. Furthermore, the left caudate, as part of a network of areas responsive to linguistic stimuli, is influenced by multilingual experience in determining the language membership of word forms [81]. This bottom-up influence highlights the LC’s role in language selection and monitoring, aligning with previous findings that emphasize its involvement in subcortical language control in bilingual individuals [82].

While our study provides valuable insights into the effects of multilingual proficiency on neurodevelopmental dynamics, several limitations merit consideration. A key limitation is the relatively small sample size, which may restrict the generalizability of our findings. A limited sample may fail to fully capture the variability present in a broader population, constraining the extent to which our conclusions can be applied to other groups or settings. Furthermore, the longitudinal design, although advantageous for examining dynamic changes, involved only two measurement timepoints, which may not adequately capture the complexity of ongoing neurodevelopmental adaptations and the trajectory of these changes over time. Future research with larger sample sizes and more frequent data collection, including participants from diverse linguistic backgrounds, would enhance our understanding of the broader applicability of the “dual” neural adaptation hypothesis across various contexts. Despite these limitations, the longitudinal aspect of the study and the integration of structural and functional neuroimaging methods represent a significant methodological strength, providing a unique opportunity to explore functional activity, connectivity, and structural plasticity over time, as well as a more holistic view of the multilingual neurodevelopmental “interactions” with cognition.

## 5. Conclusions

Taken together, the presented findings corroborate our hypothesis that a critical neurodevelopmental period for multilingual experience significantly shapes the neurobiological dynamics of brain structural and functional adaptation during childhood. The observed patterns of adaptation and specialization provide supportive evidence for the delicate interplay between experience-dependent and experience-expectant processes in inducing bilingual neuroplasticity during the consolidation phase. This phase is characterized by subcortical adaptations and enhanced efficiency in language control mechanisms, as proposed by the Dynamic Restructuring Model [20].

## Figures and Tables

**Table 1:** Sociolinguistic measures characterizing sample multilingual experience.

Language	Daily Activities: Watch TV	Daily Activities: Listen to Radio	Daily Activities: Family Conversation	Daily Activities: Conversation with Friends	Daily Activities: Read	Daily Activities: Hobbies	Exposure: Language Production	Exposure: Language Comprehension	Linguistic Competence Level	Linguistic Competence: Language Production	Linguistic Competence: Language Comprehension
German	2.4	0.6	10.2	8	6.7	3.3	3.3	3.4	L1	4	4
Italian	2.1	0.5	6.9	4.5	1.5	2.2	2.8	3.2	L2	3.2	3.6
English	0.4	1.4	0.3	0.7	0.5	1.0	1.4	1.5	L3	2.07	2.2
Ladin	0.0	0.0	0.0	0.7	0.0	0.3	1.2	1.2	L4	1.2	1.6

**Table 2:** Summary of brain regions that exhibited a negative correlation between changes in global multilingual competence and conflict effect-related BOLD activity.

Region	Hemisphere	MNI Coordinates		
		x	y	z
Caudate	Left	−18	8	18
DLPFC	Left	−42	40	22
Inferior Frontal Gyrus (pars triangularis)	Left	−32	−30	14
Putamen	Left	−22	0	6
Occipital Fusiform	Left	−36	−79	−14
DLPFC	Right	38	44	24

**Table 3:** Summary of Interaction Effect Between Differences in Left Caudate Gray Matter Volume and multilingual competence on fCE BOLD Activity (Left Caudate) between T2 and T1.

Variable	N	Mean	Std. Deviation	Minimum	Maximum	Interaction Coefficients
Intercept	-	-	-	-	-	−0.643
Delta Left Caudate (milliliters) (T2 − T1)	15	−0.036	0.080	−0.132	0.132	Independent Term: −24.451
Delta Multilingual Competence (T2 − T1)	15	−0.142	0.464	−1.300	0.820	Moderator Term: −26.272
Interaction Term on fCE BOLD Activity (T2 − T1)	15	3.04	15.60	−22.05	30.21	Interaction Term: −231.304

**Table 4:** MNI coordinates and cluster size of brain regions showing differential functional connectivity seeded from the dACC/preSMA and LC as a function of changes in multilingual competence.

dACC/preSMA + LC	MNI Coordinates			
Region	x	y	z	Cluster Size
Left Supramarginal Gyrus	−58	22	26	18
Left Cerebellum	8	−60	−48	58
Right Inferior Frontal Gyrus (Pars Triangularis)	38	34	2	56
Right Insula/Angular Gyrus	44	−54	28	68
**dACC/preSMA vs. LC**				
Region	x	y	z	Cluster Size
Left Dorsolateral Prefrontal Cortex	−18	66	26	73
Right Dorsolateral Prefrontal Cortex	60	28	22	45
Right Angular Gyrus	56	−54	28	76
Right Pre-supplementary Motor Area	6	2	52	54
**LC vs. dACC/preSMA**				
Region	x	y	z	Cluster Size
Left Fusiform Gyrus/Posterior Inferior Temporal Gyrus	−32	−48	20	38
Left Hippocampus	−20	−16	−14	13
Left Parahippocampal Gyrus	−22	−32	−28	29
Left Putamen	−22	8	2	14
Left Middle/Superior Frontal Gyrus	−24	8	60	26
Right Cerebellum	26	−32	−34	13

## Data Availability

The data presented in this study are available on request from the corresponding authors due to the involvement of a vulnerable group and confidentiality reasons.
